# Can Serum Tenascin-C Be Used as a Marker of Inflammation in Patients with Dilated Cardiomyopathy?

**DOI:** 10.1155/2013/608563

**Published:** 2013-09-09

**Authors:** Alyaa A. Kotby, Manal M. Abdel Aziz, Waleed M. El Guindy, Amira N. Moneer

**Affiliations:** ^1^Department of Pediatrics, Faculty of Medicine, Ain Shams University, Cairo 11231, Egypt; ^2^Department of Clinical Pathology, Faculty of Medicine, Ain Shams University, Cairo, Egypt

## Abstract

*Background*. Tenascin-C (TN-C) is an extracellular matrix glycoprotein that appears at sites of inflammation in cardiac pathologies. *Aim of the Work*. To evaluate the role of TN-C as a marker for active inflammation in children with dilated cardiomyopathy (DCM). *Subjects and Methods*. 24 consecutive patients with primary nonfamilial DCM aged 6–72 months (mean 45.19 ± 11.03) were divided into group I, twelve patients with acute onset DCM (<6 months duration), and group II, twelve patients with chronic DCM (>6 months duration), and compared to 20 healthy age- and sex-matched controls. Investigations included estimation of serum TN-C and echocardiographic evaluation using M-mode and 2D speckle tracking echocardiography (STE). *Results*. Serum TN-C showed a higher significant statistical elevation among patients than controls (*P* < 0.001) and in group I than group II (*P* < 0.001). EF was significantly decreased, and LVEDD and EDV increased in patients than controls and in GI than GII. STE showed a statistically significant difference in global peak strain longitudinal (GPSL) average in patients than controls (*P* < 0.05) and between GI and GII (*P* < 0.001). STE wall motion scoring showed normokinesia (33.5%), hypokinesia (8.33%), and akinesia (50%) in GI and hypokinesia (100%) in GII. There was a statistically significant positive correlation between serum TN-C and GPSL average. *Conclusions*. Increased serum TN-C can be used as a marker of inflammation in DCM and is associated with the severity of heart failure and LV dysfunction as detected by STE.

## 1. Introduction

Myocardial inflammatory diseases are an important cause of dilated cardiomyopathy (DCM) in children. Epidemics of viral myocarditis have been reported, particularly Coxsackie B virus, and the enteroviruses which are considered to be the most common cause of viral myocarditis [1]. Thus infants and young children may be more prone to develop myocarditis, due to the higher rate of enteroviral and adenoviral infections in this age group [2]. Although myocarditis has previously been speculated to account for most instances of DCM, it is now clear that 20–30% may be due to familial or genetic forms of DCM [3]; yet myocarditis might still be implicated in the initiation of the process. 

There are few biomarkers for myocarditis. Full blood count and erythrocytic sedimentation rate are not usually helpful. Troponin I has a high specificity for diagnosing myocarditis but a sensitivity of only 34%, and creatine kinase and its cardiac isoform CK-MB are less sensitive and specific than troponin [4]. Increased levels of autoantibodies against myocardial proteins have also been reported [5].

Tenascin-C (TN-C) is an extracellular matrix glycoprotein [6] that is not expressed in normal adult hearts but is expressed in various myocardial diseases such as acute myocarditis [7–9], dilated cardiomyopathy [10], myocardial infarction [11], and myocardial hibernation [12].

Speckletracking echocardiography (STE) is a noninvasive ultrasound imaging technique that allows for an objective and quantitative evaluation of global and regional myocardial function independently from the angle of insonation and from cardiac translational movements [13]. Its advantage over conventional echo warrants its use in the assessment of the global and regional myocardial performance in acute and chronic DCM.

We hypothesized that serum TN-C might serve as a marker of active myocardial inflammation in children with new onset DCM.

## 2. Subjects and Methods

This case controlled study was conducted in the Pediatric Cardiology Clinic and Echocardiography Laboratory, Children's Hospital, Ain shams University. The study included 24 consecutive patients with DCM aged 6–72 months with a mean age of 45.19 ± 11.03 months and 20 healthy children. The subjects were divided into 3 groups: group I included twelve patients with acute DCM of less than 6 months duration; group II included twelve patients with chronic DCM for more than 6 months; control group included twenty (20) healthy age- and sex-matched children. The control group was chosen from healthy children who came with their sick siblings to the outpatient clinic of the children's hospital.

Clinical diagnosis of DCM was based on the WHO [14] and American Heart Association [15] criteria. At time of diagnosis all patients had an ejection fraction <45% and/or a fractional shortening of <25% and a left ventricular end diastolic dimension (LVEDD) of >112% of the predicted value corrected for age and body surface area [14]. All patients received antifailure measures in the form of inotropes (lanoxin), diuretics (furosemide and spironolactone), and angiotensin enzyme inhibitor (captopril). Patients with familial, genetic, or secondary cardiomyopathy were excluded. A written informed consent was given by parents of patients and controls, and the study protocol was approved by the institutional review board.

*Methods. *All studied patients were subjected to full medical history, thorough clinical examination, and investigations that included the following.

*(i) Laboratory. *Serum TN-C was measured. Serum levels of TN-C with the large subunit containing the C domain of FITIIII repeats were determined using an ELISA technique with 2 monoclonal antibodies, 19C4MS and 4F10TT (IBL, Gunma, Japan) [16].

*(ii) Echocardiography*. Echocardiography was done. M-mode, two dimensional,continuous, pulsed colors Doppler and Speckle track echocardiography (STE) transthoracic echocardiography was done using device model GE medical system VIVID7 dimension N-3190, Horten, Norway. STE was performed according to the methods recommended by the American Society of Echocardiography [17]. Longitudinal strain (LS) was assessed in standard four-chamber, three-chamber (apical long-axis), and two-chamber apical views. The images were obtained at end-expiratory phase using a 5 MHz center frequency phased-array probe with second-harmonic imaging. The settings were configured to obtain optimal quality images. Frame rates were kept between 60 and 90 frames/sec. They were stored in cine loop format for offline analysis by vendor customized software (Echo PAC PC-2D Strain; GE Medical Systems). LS was measured in 16 LV segments by tracing the endocardial contour on an end-systolic frame that allowed the software to automatically place the contour on subsequent frames by temporally tracking the “natural acoustic speckle” in the B-mode images. Full thickness of myocardium from endocardial to epicardial borders was covered. Adequate tracking for the study was verified in real time, and in segments with poor tracking, the endocardial trace line was readjusted until a better tracking score was achieved. A suboptimally tracked segment was excluded from further analysis. LS curves reflected the average value of all of the acoustic markers in each segment [17].

*(iii) Statistical Analysis*. Data were expressed as means ± SD for continuous variables and as numbers (percentages) for categorical variables. Continuous variables were analyzed by the unpaired Student's *t*-test. Pearson's or Spearman's correlation analysis was performed to estimate correlations between variables. A *P* value <0.05 was considered statistically significant.

## 3. Results

The levels of TN-C of all patients (groups I and II) and controls are shown in Tables 1 and 2.

M-mode echocardiographic data of the patients in comparison to the control group are shown in Table 3 and those of group I in comparison with group II are shown in Table 4.

**Figure 1:**
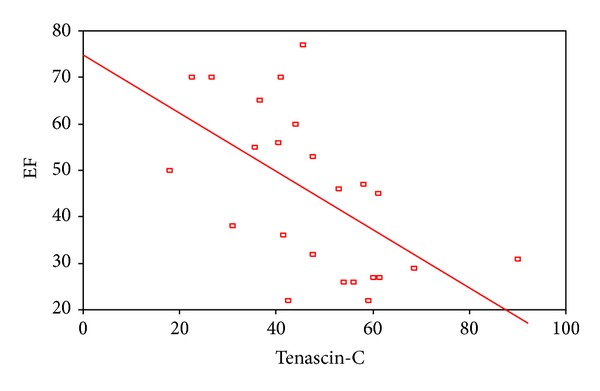
A negative correlation between serum tenascin-c level and ejection fraction in patients.

**Figure 2:**
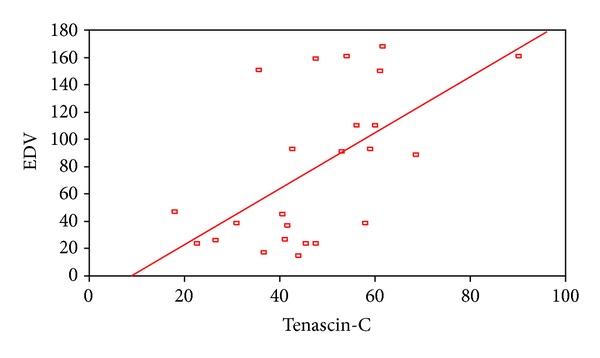
A positive correlation between serum tenascin-c and end diastolic volume in patients.

**Figure 3:**
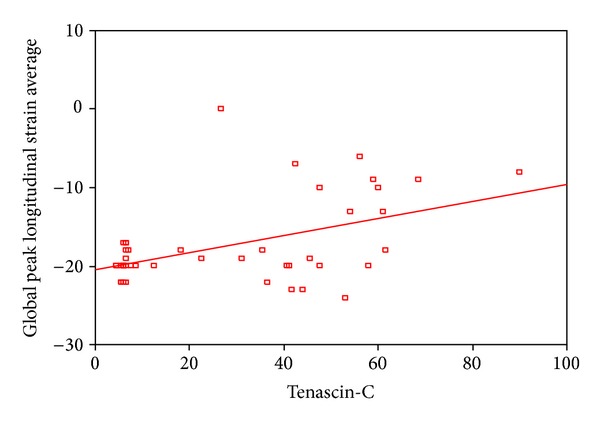
A significant positive correlation between serum tenascin-c level and global peak longitudinal strain average in DCM patients.

**Figure 4:**
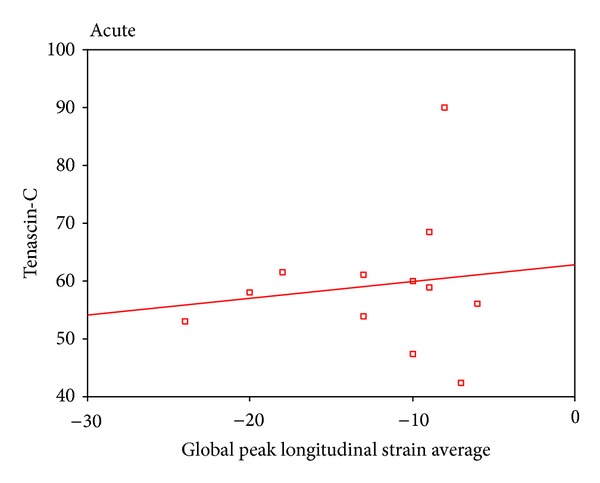
A significant positive correlation between serum tenascin-c and global peak longitudinal strain average in group I.

**Figure 5:**
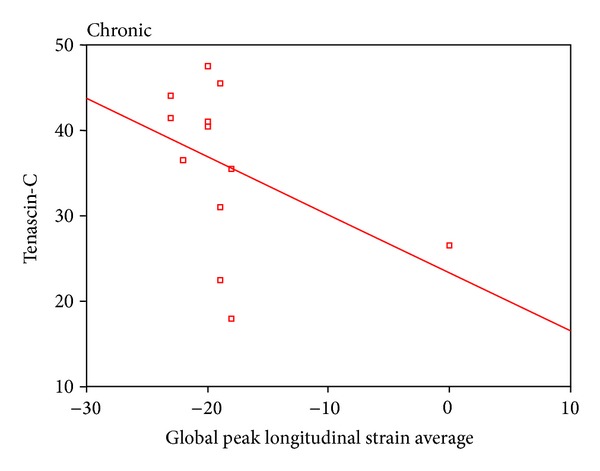
An insignificant negative correlation between serum TN-C and global peak longitudinal strain average in group II.

2D STE showed that there was a highly statistical significant difference between patients and controls as regard global peak strain longitudinal apical long axis (G SL ap lax), G peak SL apical 4 chambers (a4c), G peak SL apical 2 chambers (a2c), and G peak SL average. STE of groups I and II is shown in Table 4. STE wall motion score was normokinetic in all controls while data of groups I and II are shown in Table 5. Correlation between serum TN-C level and the echocardiographic findings showed a statistically significant negative correlation between serum tenascin-c and EF in all patients (Figure 1) and a statistically significant positive correlation between serum TN-C level and LVEDD and enddiastolic volume in patients (Figure 2) and in acute cases. Correlation between serum TN-C level and patients, group I and group II, is shown in Figures 3, 4, and 5, respectively.

## 4. Discussion

Serum TN-C levels have not been previously studied in pediatric patients with DCM. In the present study, we attempted to investigate the utility of its use as a marker of inflammation in infants and children with DCM. To avoid noninfectious causes of DCM, patients with familial/genetic or secondary cardiomyopathy were excluded. We found serum TN-C levels to be significantly higher in children with DCM than those in controls and in acute than chronic DCM. The significant elevation of TN-C in group I might be due to an inflammatory process since children at this age group are more prone to develop DCM following viral myocarditis [2]. Previous data demonstrated that TNC is a useful marker for evaluation of disease activity in myocarditis [7, 9]. In their study on adult myocardial samples Tsukada et al. [18] found a high prevalence of chronic myocarditis in DCM patients and suggested that TN-C might prove to be a useful marker for distinguishing inflammatory cardiomyopathy from other types of DCM. Researchers found that most of the myocardium in DCM patients shows varying degrees of inflammation and that expression of TN-C is enhanced in the areas of active inflammation with local tissue remodeling [19].

Serum TN-C correlated negatively with the EF and positively with the LVIDD and EDV suggesting that a high serum TN-C associated the impaired myocardial functions. Similarly, Aso et al. [10] found that serum TN-C levels were increased in proportion to the severity of left ventricular dysfunction in patients with IDC. The decrease in TN-C that associated the improved EF in group II is suggestive of an improvement but not disappearance of the inflammatory process in chronic DCM. Yet the role of ACEI in blocking vascular TN-C expression cannot be excluded as our patients with chronic DCM were maintained on ACEI. Angiotensin II is a potent inducer of tenascin-C, with drugs such as angiotensin II type 1 receptor (AT-1) antagonists, and angiotensin converting enzyme (ACE) inhibitors potentially block vascular tenascin-C expression in hypertensive patients [20]. On the other hand, we cannot exclude the role of LV dysfunction and heart failure in increasing serum TN-C in our study group. It was suggested that the increase in serum TN-C levels was associated with the severity of heart failure and LV dysfunction and remodeling in patients with DCM [10, 19]. Owing to the significant correlation between the TN-C level and LVDD, it was suggested as a new biomarker for detecting cardiomyopathy in patients with Emery-Dreifuss muscular dystrophy [21]. 

The recently introduced STE allows easy assessment of segmental and global longitudinal LV function and provides information on top of ejection fraction [13]. The significant positive correlation between serum TN-C and global peak longitudinal strain average in patients indicates that the increase in serum TN-C levels was associated with deterioration in cardiac function as detected by STE. We used the global longitudinal strain since it has been demonstrated that it is a more robust parameter than radial and circumferential strain for the assessment of myocardial function [22]. The STE wall motion scores in group I showed that 33.5% were normokinetic, 8.33% were hypokinetic, and 50% were akinetic. These data are suggestive of the prognosis in this group, where 1/3 of patients with acute DCM are apt to have improved LV functions after the inflammatory process subsides while those with akinesia might either improve or die. STE was more sensitive than conventional echocardiography in detecting wall motion abnormalities in group II, where all the patients had hypokinetic wall motion scoring by STE inspite of the normalized EF. This indicates an ongoing process of myocardial affection in chronic DCM that is associated by a raised serum TN-C.

Drugs targeting the expression or function of tenascin-C or the tenascin-C protein itself are currently being developed [23]. They might help in the better management of acute and chronic cardiomyopathy of infectious origin. 

## 5. Study Limitations

After the exclusion of the familial/genetic and secondary causes of DCM, the number of patients was small. Once diagnosed all patients received angiotensin converting enzyme inhibitors which are known to block vascular TN-C expression.

## 6. Conclusions

Serum-TN-C levels are increased in children with DCM and can be used as a marker of inflammation in acute cases. Its persistence in chronic DCM might lead to progressive myocardial disease and ventricular dilation. STE is more sensitive than conventional echocardiography in the assessment of myocardial performance in DCM. Its values correlate with serum TN-C. Drugs targeting the expression or function of tenascin-C might offer new perspectives for therapeutic approaches in this specific population.

## Figures and Tables

**Table 1:** A highly significant statistical difference between patients and controls as regard serum tenascin-C level.

Tenascin-C (ng/mL)	DCM patients (groups I and II)	Controls
Range	18–90	4.5–12.5
Mean	47.45	6.50
±SD	15.89	1.67
*t*-test	11.472	
*P* value	<0.001 highly significant	

**Table 2:** Serum tenascin-C levels in groups I and II.

Tenascin-C (ng/mL)	Group I	Group II
Range	42.5–90	18–47.5
Mean	59.25	35.83
±SD	11.82	9.45
*t*-test	5.358	
*P* value	<0.001 highly significant	

**Table 3:** M-mode echocardiographic data of patients compared to controls.

M-mode		*N*	Mean	±SD	*t*-test	*P* value
LVIDD (cm)	Patients	24	3.84	1.18	4.860	0.001**
	Control	20	2.52	0.29		

LVIDS (cm)	Patients	24	3.13	1.24	4.223	0.001**
	Control	20	1.95	0.06		

EDV (mL)	Patients	24	79.16	55.25	3.645	<0.05*
	Control	20	33.90	2.26		

ESV (mL)	Patients	24	50.79	42.29	3.806	0.001**
	Control	20	14.70	1.78		

EF%	Patients	24	45	17.46	4.385	0.001**
	Control	20	62.25	2.44		

FS%	Patients	24	22.91	10.72	3.855	0.001**
	Control	20	32.25	1.40		

LA/AO	Patients	24	1.78	0.88	2.006	>0.05
	Control	20	0.88	0.30		

**Highly significant, *Significant. LVIDD: left ventricular internal diameter in diastole, LVIDS: left ventricular internal diameter in systole, EF: ejection fraction, FS: fraction shortening, AO: aortic annulus, LA: left atrium, EDV: end-diastolic volume, ESV: end-systolic volume, LA: left atrium, and Ao: aorta.

**Table 4:** M-mode echocardiographic data of group I compared to group II.

M-mode		*N*	Mean	±SD	*t*-test	*P* value
LVIDD (cm)	Group I	12	4.62	0.91	4.264	0.001**
	Group II	12	3.06	0.87		

LVIDS (cm)	Group I	12	4.10	0.79	5.997	0.001**
	Group II	12	2.17	0.77		

EDV (mL)	Group I	12	118.6	40.5	5.013	0.001**
	Group II	12	39.66	36.5		

ESV (mL)	Group I	12	84.41	30.9	6.528	0.001**
	Group II	12	17.16	17.8		

EF%	Group I	12	31.66	9.14	5.841	0.001**
	Group II	12	58.33	12.90		

FS%	Group I	12	14.75	4.18	5.799	0.001**
	Group II	12	31.08	8.81		

LA/AO	Group I	12	2.20	1.08	2.558	<0.05*
	Group II	12	1.37	0.26		

**Highly significant, *Significant. LVIDD: left Ventricular internal diameter in diastole, LVIDS: left ventricular internal diameter in systole, EF: ejection fraction, FS: fraction shortening, AO: aortic annulus, LA: left atrium, EDV: end-diastolic volume, ESV: end-systolic volume, LA: left atrium, and Ao: aorta.

**Table 5:** G-peak sl ap lax, G peak sl a2c, G peak sl a4c, and G peak sl avg in group I and group II.

Tissue		*N*	Range	Mean	±SD	*t*-test	*P* value
G peak sl ap lax	Group I	12	−7–−20	−12.08	5.36	4.922	0.001**
	Group II	12	−16–−24	−20.50	2.50		

G peak sl a4c	Group I	12	−4–−20	−11.91	5.35	2.042	<0.05*
	Group II	12	−10–−23	−15.41	2.574		

G peak sl a2c	Group I	12	−9–−19	−14	6.53	4.227	0.001**
	Group II	12	−15–−25	−24.2	5.27		

Peak sl avg	Group I	12	−6–−19	−12.25	5.62	2.585	<0.05*
	Group II	12	−18–−24	−18.41	6.05		

**Highly significant, *Significant.
